# Modulation of Pulse Propagation and Blood Flow via Cuff Inflation—New Distal Insights

**DOI:** 10.3390/s21165593

**Published:** 2021-08-19

**Authors:** Laura I. Bogatu, Simona Turco, Massimo Mischi, Lars Schmitt, Pierre Woerlee, Erik Bresch, Gerrit J. Noordergraaf, Igor Paulussen, Arthur Bouwman, Hendrikus H. M. Korsten, Jens Muehlsteff

**Affiliations:** 1 Department of Electrical Engineering, Eindhoven University of Technology, 5612AZ Eindhoven, The Netherlands; s.turco@tue.nl (S.T.); m.mischi@tue.nl (M.M.); p.h.woerlee@tue.nl (P.W.); 2 Philips Research, 5656AE Eindhoven, The Netherlands; lars.schmitt@philips.com (L.S.); erik.bresch@philips.com (E.B.); igor.paulussen@philips.com (I.P.); jens.muehlsteff@philips.com (J.M.); 3 Elisabeth-TweeSteden Hospital, 5022GC Tilburg, The Netherlands; g.noordergraaf@etz.nl; 4 Catharina Ziekenhuis, 5623EJ Eindhoven, The Netherlands; arthur.bouwman@catharinaziekenhuis.nl (A.B.); erik.korsten@catharinaziekenhuis.nl (H.H.M.K.)

**Keywords:** pulse transit time, pulse arrival time, cuff-based measurement, blood pressure cuff, functional hemodynamic monitoring, occlusion-based perturbation, pulse propagation modulation, flow modulation

## Abstract

In standard critical care practice, cuff sphygmomanometry is widely used for intermittent blood pressure (BP) measurements. However, cuff devices offer ample possibility of modulating blood flow and pulse propagation along the artery. We explore underutilized arrangements of sensors involving cuff devices which could be of use in critical care to reveal additional information on compensatory mechanisms. In our previous work, we analyzed the response of the vasculature to occlusion perturbations by means of observations obtained non-invasively. In this study, our aim is to (1) acquire additional insights by means of invasive measurements and (2) based on these insights, further develop cuff-based measurement strategies. Invasive BP experimental data is collected downstream from the cuff in two patients monitored in the OR. It is found that highly dynamic processes occur in the distal arm during cuff inflation. Mean arterial pressure increases in the distal artery by 20 mmHg, leading to a decrease in pulse transit time by 20 ms. Previous characterizations neglected such distal vasculature effects. A model is developed to reproduce the observed behaviors and to provide a possible explanation of the factors that influence the distal arm mechanisms. We apply the new findings to further develop measurement strategies aimed at acquiring information on pulse arrival time vs. BP calibration, artery compliance, peripheral resistance, artery-vein interaction.

## 1. Introduction

In standard hemodynamic monitoring practice, the cuff measurement principle consists of altering the transmural pressure across the brachial arterial wall; the resulting brachial arterial volume oscillations are measured and interpreted via an empirical method to estimate blood pressure (BP). This measurement is performed at intervals between five minutes to several hours depending on the patient’s condition.

A hypothesis is that the cuff is underutilized in clinical practice and that other measurement strategies can be developed based on occlusion-based modulation of flow and pulse propagation along the artery, in combination with standard monitoring equipment (e.g., electrocardiogram ECG, photoplethysmogram PPG). For this reason, in our previous work we explored how to adapt the cuff measurement principle to obtain further information, in addition to the intermittently acquired BP values.

- In [1], the cuff is used to alter the transmural pressure across the brachial artery for the purpose of modulating Pulse Arrival Time (PAT). The change in PAT with respect to the controlled transmural pressure is analyzed in order to calibrate the BP-PAT relationship for the purpose of beat-to-beat PAT-based BP estimation.
- In [2] both the brachial artery volume oscillation and the PAT response to cuff-based perturbation are interpreted in order to estimate brachial arterial compliance and collapse mechanics. Changes in these vascular properties can precede changes in BP and cardiac output [2]. Intensive care clinicians have expressed interest in measuring short-term, dynamic regulation of arteries [3], however, methods for detecting such regulation mechanisms are not yet available, mainly due to the difficulty of measuring vascular properties with standard hemodynamic monitoring hospital equipment.

The two studies [1,2] involved modelling of the localized change in transmural pressure and its impact on pressure pulse propagation: as the cuff inflates, the arterial transmural pressure over the length of the cuff is decreased, thus increasing the time it takes for the pulse to propagate down the brachial artery. These studies demonstrated this effect by means of non-invasive measurement setups (consisting of BP cuff, ECG, PPG), complemented by computer simulations.

Due to the noisy nature of the non-invasive measurements, it was not possible to thoroughly study the effects occurring downstream from the cuff. This led to the development of a model which characterized the brachial vasculature, but excluded effects occurring in the distal limb vasculature (under the assumption that distal BP and distal pulse transit time do not change significantly throughout cuff inflation) [1,2]. Other previous studies involving cuff-based modulation of PAT also lack experimental characterization of distal vasculature effects, leading to unverified assumptions and development of unrealistic models [4,5]. In general, numerous studies tackle the brachial artery collapse caused by cuff inflation (for example, via ex-vivo studies [6], ultrasound measurements [7], computer simulations [8]), however, effects of cuff inflation on the distal artery are not being thoroughly investigated. To further advance our understanding of vasculature response to occlusion-based perturbations, in this study we measure blood pressure downstream from the cuff invasively in two patients monitored in the operating room (OR). We observe highly dynamic and patient specific effects occurring in the distal arm as a result of cuff inflation. The observations lead to improved interpretation of the cuff measurement. We develop a model in order to reproduce the observed distal vasculature behavior. We apply the new findings to improve the measurements presented in [1,2]. In addition to this, we demonstrate that information on parameters such as distal arterial compliance, artery-vein interaction, mean systemic filling pressure, changes in arm peripheral resistance (possibly representative of changes in systemic resistance), can be extracted from the distal BP response to cuff inflation. The presented measurement strategy, data and model-based analysis are aimed at establishing a basis for further investigations of occlusion-based perturbations applied to the circulation and of the resulting activation of compensatory mechanisms.

## 2. Materials and Methods

### 2.1. Measurement Procedure

**Figure 1:**
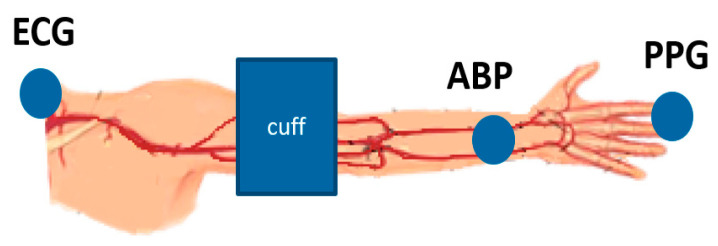
Sensor arrangement consisting of electrocardiogram ECG, blood pressure cuff, radial intra-arterial line ABP and photoplethysmogram PPG.

A sensor arrangement consisting of ECG, brachial BP cuff, radial intra-arterial line (ABP) and finger PPG (Figure 1) is used to collect data from two anesthetized and mechanically ventilated patients undergoing non-cardiac surgery. All sensors are standard devices common in clinical practice: Philips Comfort Care Adult Cuff [9], Edwards Lifesciences TruWave disposable pressure transducer (Edwards Lifesciences, Irvine, CA, USA) [10] and Philips PPG model M1191B [9].

The data is recorded using a Philips MP50 patient monitor [9] and custom data logging software. The ECG is sampled at 500 Hz, and the ABP, PPG, and cuff pressure is sampled at 125 Hz. The data collection is approved by the MEC-U ethical committee (St. Antonius Ziekenhuis, Koekoekslaan 1, 3430 EM Nieuwegein, NL. Approval W19.046) and the data is collected at the Elisabeth-TweeSteden Ziekenhuis, Tilburg, NL. Written informed consent was obtained from the patients. For reasons of clarity, the two patients are distinguished as $Patient_{1}$ and $Patient_{2}$. For both patients, cuff inflations are performed at arbitrary intervals across 7 h before, during and after a surgical procedure. Cuff pressure, ECG, ABP and PPG signals are simultaneously recorded during cuff inflations. Changes in PAT and pulse transit time (PTT) due to cuff inflation are computed over two vascular segments:

- Heart to finger site: $\Delta PAT_{ECG-PPG}\left(CuffP\right)$ is computed as the change in delay between the R-peak of the ECG signal and the foot of the PPG waveform as cuff pressure CuffP increases.
- Radial to finger site: $\Delta PTT_{ABP-PPG}\left(CuffP\right)$ is computed as the change in delay between the foot of the ABP waveform and the foot of the PPG waveform as cuff pressure CuffP increases.

Note that invasive surgical procedures are performed on the patients, abrupt hemodynamic alterations are induced and motion artifacts are present in the signals. For this reason, 12 out of 48 cuff inflations had to be removed from *Patient*_1_ data. 21 out of 68 cuff inflations had to be removed from *Patient*_2_ data. Removal criteria include presence of motion artifacts causing the pulse to be indistinguishable over portions of the signal, flushing of the arterial line, pressure pulse change greater than 20 mmHg at 20 s after the cuff inflation compared to the start of the cuff inflation (indicative of significant hemodynamic events, which hider the analysis of cuff-induced effects). Throughout each cuff inflation, changes in ABP and in distal PTT are observed.

### 2.2. Model

A model is developed to identify the factors that influence the observed dynamic behaviors. The model is then used to further develop the methods presented in [1,2] and to explore opportunities for deriving additional hemodynamic parameters.

The model presented by Seagar et al. in [11] was adapted for our experimental condition. The study in [11] was aimed at the detection of venous thrombosis; patient data on limb volume and venous pressure was collected at the distal side of a cuff while the cuff was held inflated at a constant pressure to stop the blood flow out of the limb. The data was then fitted via a parametric model of the limb circulation; the model parameters were representative of clinically relevant indices related to thrombosis. The model however included parameters such as systemic resistance and blood vessel compliance, which are also relevant to hemodynamic monitoring, this fact being stated by the author: *“… a particularly appropriate application for the model is to use changes in the model parameters to monitor circulatory changes of the limb, such as those, for instance, that may occur during clinical anaesthesia”* [11].

**Figure 2:**
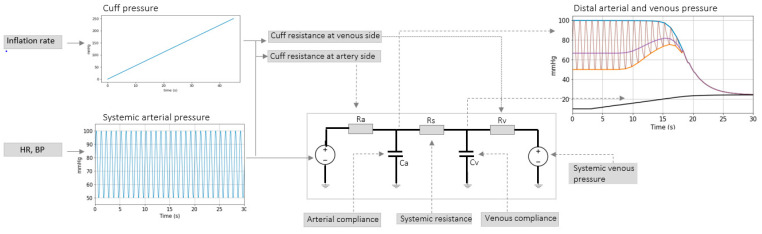
Overview of arm circulation model including a change in $R_{a}$ due to cuff inflation.

The venous thrombosis model in [11] is therefore adjusted to characterize our critical care data. Figure 2 shows an overview of the adapted model. Systemic arterial pressure, cuff pressure and several parameter values are considered as input to the model. The model outputs are the distal arterial pressure and distal venous pressure. $R_{a}$ represents the resistance to blood flow over the brachial artery. Its value is estimated via the Poiseuille Equation,

(1) $$R_{a}\left(P_{tm}\right)=\frac{8\eta L}{\pi \;r{\left(P_{tm}\right)}^{4}}\;$$

where η is the blood viscosity, *L* is the length cuff length and $r\left(P_{tm}\right)$ is brachial-artery radius as function of transmural pressure $P_{tm}$ across the arterial wall:

(2) $$P_{tm}=P_{art}–\;P_{cuff}$$

where $P_{art}$ is the arterial pressure under the cuff. Physiological ranges of absolute value of radius r is obtained from ultrasound measurement of brachial artery performed by Bank et al. in [7].

As cuff pressure $P_{cuff}$ increases, the artery radius decreases as function of transmural pressure. The function is defined via the equation describing arterial collapse mechanics from [2,6]:

(3) $$A\left(P_{tm}\right)=d\;\frac{\ln\left(a\;Ptm+3.3\right)}{1+e^{-c\;P_{tm}}}$$

where *A* is arterial cross sectional area and *a*, *c*, *d* are parameters describing arterial collapse. The resulting $R_{a}$ (illustrated in Figure 2) as function of transmural pressure is proportional to the fourth power of the radius. $R_{v}$ represents the resistance to blood flow over the brachial vein. During fast inflations $R_{v}$ does not influence the arterial BP behavior significantly—evidence on vein collapse behavior ([12,13], including MRI images collected as part of our previous studies [14,15]) confirm that the vein collapses at approximately −10 mmHg venous transmural pressure. The physiologic venous pressure range is between 5 mmHg and 15 mmHg [12], from which it can be concluded that the vein collapses in the very first part of the inflation (before cuff reaches 30 mmHg).

$R_{s}$ represents the arm peripheral resistance. Physiological ranges for peripheral resistance in the arm were obtained from a study aimed at assessing hemodialysis access sites [16]. The study reports direct measurements of resistance via flow rate and pressure acquired in-vivo in the upper arm. Resistance values of about 100 mmHg·s/mL are reported. *C_a_* and *C_v_* represent arterial and venous compliance respectively. In [16] arterial compliance *C_a_* in the arm is approximated in the order of 0.03 ${\mathrm{mL}\;\mathrm{mmHg}}^{-1}$. *C_v_* is approximated as about 30 times larger than *C_a_* [17]. 

Simulations conducted via the model (Figure 2) with parameters within ranges close to the reported values mimic effects observed in the patient data, showing that the model can be used to represent changes in distal arterial pressure during cuff inflation and that some physiological meaning can be attributed to the model parameters. 

### 2.3. Parameter Inference

The model is used to:

- Improve the inference method of brachial artery compliance presented in [2].
- Assess the estimation of PAT-BP calibration by further development of the method presented in [1].
- Infer distal arm circulation parameters.

#### 2.3.1. Brachial Artery Compliance Inference 

In [2], a method to obtain information on smooth muscle tone/compliance of the artery located under the cuff is proposed. The method involves modulation of pulse propagation along the arm via inflation of a cuff placed at the brachial site. The resulting PTT with respect to cuff inflation is processed to obtain the parameters *a* and *c* describing the brachial artery volume with respect to arterial transmural pressure $V_{art}\left(P_{tm}\right)$. $V_{art}\left(P_{tm}\right)$ is expressed via the brachial artery area (Equation (3)) multiplied by the cuff length. 

PTT was measured via ECG and finger PPG. The method assumed that the only portion of the artery where changes in PTT occur are along the length of the cuff, and that the transit time in the distal artery remains unaltered during cuff inflation. This assumption was based on preliminary results involving non-invasive measurements. Our new invasive measurements, however, reveal that significant changes do occur in the distal artery.

##### Simulation Framework

To improve the method presented in [2], a simulation framework is used. A distal BP signal is generated according to the model in Figure 2. Systemic arterial diastolic pressure is set to 50 mmHg and systemic arterial systolic pressure is set to 100 mmHg. Heart rate is set to 1 Hz and cuff inflation speed is set to 25 s. The model parameters are chosen based on the values reported in the literature: systemic venous pressure is set to 10 mmHg [12], peripheral resistance *R_s_* is 106 mmHg·s/mL [16], arterial compliance *C_a_* is 0.03 mL/mmHg [16], venous compliance *C_v_* is 0.9 mL/mmHg [17]. BP-PWV relationship is expected to be approximately linear over transmural pressure regions above 50 mmHg [6,7]. Therefore, a linear relationship between distal MAP and pulse wave velocity (PWV) is fitted to obtain PTT changes of similar amplitudes as the ones observed in the patient data.

The simulated signals are analyzed in order to assess the impact of distal artery effects on the correctness of the inference process described in [2] (which assumed that no change takes place in distal PTT) and to investigate mitigation solutions. 

##### Inference of Brachial Artery Parameters from Patient Data

The findings obtained at point (*a*) are applied to the patient data in order to infer brachial artery compliance. Over the course of each inflation, changes in PTT occurring over the length of the cuff ($\Delta PTT_{Brachial}$) are obtained via Equation (4):

(4) $$\Delta PTT_{Brachial}\left(CuffP\right)=\Delta PAT_{ECG-PPG}\left(CuffP\right)-2\cdot \Delta PTT_{ABP-PPG}\left(CuffP\right)$$

The drop in ΔPTT over the entire length of the distal arm is assumed to be twice the drop in $\Delta PTT_{ABP-PPG}$, due to the arterial line being placed halfway between the distal edge of the cuff and the fingertip. Note that this is a simplified model, which assumed a homogeneous artery segment. $\Delta PTT_{Brachial}$ is then processed via the inference modality described in [2], to obtain brachial artery volume with respect to arterial transmural pressure $V_{art}\left(P_{tm}\right)$ at the time of each inflation. 

#### 2.3.2. Estimation of PAT-BP Calibration from Patient Data

The inferred $V_{art}\left(P_{tm}\right)$ is used to estimate the calibration at the time of each inflation. First, the Bramwell-Hill equation (Equation (5)) is used to compute PWV with respect to $P_{tm}$.

(5) $$PWV\left(P_{tm}\right)=\sqrt{\frac{V_{art}\left(P_{tm}\right)}{\rho \times C_{art}\left(P_{tm}\right)}}$$

where $P_{tm}$ is the transmural pressure across the arterial wall, ρ is the blood density, $V_{art}\left(P_{tm}\right)$ is the arterial volume as a function of $P_{tm}$, and $C_{art}\left(P_{tm}\right)$ is the arterial compliance as a function of $P_{tm}$ (derivative of $V_{art}\left(P_{tm}\right)$). 

For a time segment between two consecutive cuff inflations, the beat-to-beat PAT value is calculated as the time delay between the R-peak of the ECG signal and the foot of the PPG signal. For each heartbeat, Equation (6) is used to estimate PWV based on the measured change in PAT.

(6) PWV=1dPATmeasuredL +1PWV(PSysRef)

where $dPAT_{measured}$ is the PAT change with respect to the reference PAT value measured at the beginning of the time segment,$\;P_{SysRef}$ is defined as the systemic systolic pressure at the moment of cuff inflation, and L is the length travelled by the pulse (heart to finger site), approximated as 1 m [1]. It is assumed that the heart pre-ejection period (PEP) does not change over the time segment. The transmural pressures corresponding to each computed PWV value is found via the $P_{tm}-\mathrm{PWV}$ relationship previously obtained via Equation (5). This resulting transmural pressure is assumed to be the systolic pressure value of the corresponding heartbeat. In this way, the beat-to-beat systolic value is estimated over the time segment between two consecutive cuff inflations. For validation, the estimated systolic value is compared to the invasively measured systolic value.

#### 2.3.3. Inference of Distal Arm Circulation Parameters

##### Time Constant and Mean Systemic Filling Pressure 

The response of BP to cuff inflation is complex; the signal contains many features from which physiological meaning could be derived. However, artifacts and interference between multiple effects are also present. The portion of the signal which is influenced by the least number of factors is the exponential decline occurring after complete arterial collapse. In this region, the signal is not influenced by cardiac activity, breathing artifacts, or by blood flow in/out of the limb. This portion of the signal is investigated first. 

The exponential decline can be characterized by two parameters: the value towards which arterial and venous pressures tend to ($P_{Equilibrium}$), and time constant (τ). When cuff inflation is very fast (when artery and vein collapse simultaneously) $P_{Equilibrium}$ can be defined as the mean systemic filing pressure *P*_msf_. *P*_msf_ is the mean pressure in the circulatory system when there is no blood in motion. This value is of great interest to clinicians because it holds information on the fluid status of a patient [18]. There is a great need for reliable *P*_msf_ measurements in the clinic. Previously investigated cuff-based *P*_msf_ measurement modalities involve complete vascular occlusion of about ~30 s to allow for venous and arterial pressures to reach an equilibrium value [19]. We investigate if the distal arm model (Figure 2) can be used to estimate *P*_msf_ via a ~5 s arterial occlusion.

The time constant τ is a non-standard parameter, but it is potentially valuable to hemodynamic monitoring as it holds information on the artery-vein interaction (the rate at which arterial and venous pressures tent towards the equilibrium value). 

To demonstrate a measurement strategy aimed at estimation of τ and $P_{Equilibrium}$, a simulation framework is used. τ and $P_{Equilibrium}$ values are inferred by fitting an exponential function to a signal generated via the model in Figure 2. Analytically derived parameter values are compared to exponential fit results.

The patient data is then processed to infer τ and $P_{Equilibrium}$ values at each cuff inflation. The results are analyzed qualitatively to identify correlations between different hemodynamic parameters.

##### Systemic Resistance and Distal Vascular Compliance

**Figure 3:**
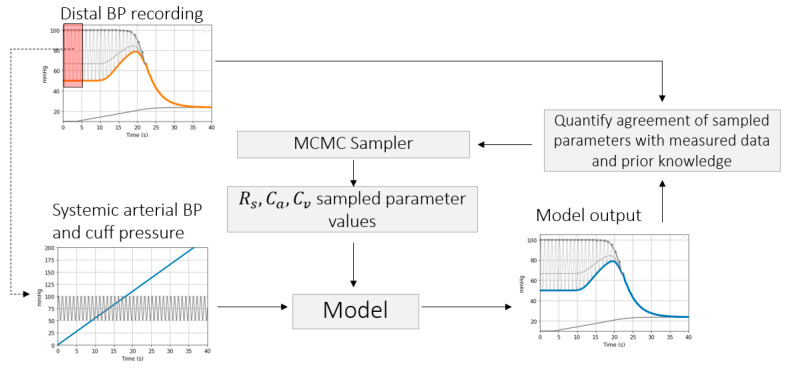
Sampling process to be repeated over a number of iterations to determine the posterior distribution of model parameters.

Several other parameters can be inferred by taking into account the complete BP signal recorded over the entire cuff-inflation duration. A dedicated estimator is introduced to infer distal circulation parameters of the model shown in Figure 3 arterial compliance *C_a_*, arm vasculature resistance *R_s_* and venous compliance *C_v_*. The algorithm is based on standard Bayes Markov Chain Monte Carlo (MCMC) processing [20]. This method is chosen due to the complex interference between multiple physiological effects affecting the signal–brachial artery collapse (influenced by parameters *a*, *c*, *d*), artery-vein interaction (parameters τ and $P_{Equilibrium}$), arterial venous collapse, heart and lung activity. Usage of prior knowledge and transparent interpretation of the inference results are needed. 

The algorithm receives as input the systemic arterial pressure waveform and cuff pressure. To determine the systemic arterial pressure throughout the cuff inflation without introducing a second invasive arterial line, it is necessary to assume that prior to the cuff inflation, the systemic arterial pressure equals the distal arterial pressure and that the systemic arterial BP stays constant throughout the cuff inflation.

A sampling process is repeated over a pre-defined number of iterations in order to represent the posterior distribution of arterial collapse parameters characterized by Equations (7) and (8). With every iteration, a set of parameter values, *C_a_*, *R_s_*, and *C_v_*, are sampled from the posterior and used as input to the model presented in Figure 3 to compute a distal arterial pressure signal. Agreement of sampled parameter values with measured data and with prior knowledge is evaluated via Equations (7) and (8). An illustration of the sampling process is shown in Figure 3.

(7) P(θ |D)∝P(θ)P(D|θ),

where P(θ |D) represents the posterior distribution, θ represents the parameter set $\left[C_{a},R_{s},C_{v}\right]$ and D represents the measured data $P_{\mathrm{cuff}}{\;\mathrm{and}\;\mathrm{BP}}_{\mathrm{env}}\left(P_{\mathrm{cuff}}\right).{\;\mathrm{BP}}_{\mathrm{env}}$ is defined as the lower envelope of the distal BP signal (illustrated in Figure 3). P(D|θ) is defined as:

(8) $$P\left(D|\theta \right)=\sum_{P_{\mathrm{cuff}}=0}^{\;\max\left(P_{\mathrm{cuff}}\right)}\log\left(\frac{1}{\sqrt{2\pi \sigma^{2}}}\;\right)–\;\frac{{\left({\mathrm{BP}}_{\mathrm{env}}\left(P_{\mathrm{cuff}}\right)-{\;\mathrm{BP}}_{\mathrm{env}\theta }\left(P_{\mathrm{cuff}}\right)\right)}^{2}}{2\sigma^{2}}$$

where σ, the expected measurement noise is set to 2 mmHg, according to measurement device specifications [10].

The resulting posterior distribution is interpreted to obtain the 95% most probable parameter values, (also known as highest density interval HDI), together with the distribution’s central tendency.

To demonstrate the feasibility of the inference method, the algorithm is run on simulated BP signals generated via control parameter sets [*C_a_*, *R_s_*, *C_v_*]. Inferred parameter values are compared against the control parameter values.

The prior P(θ) is used in the simulation framework to enforce that the parameter sets satisfy the expected physiological properties (compliance parameters cannot take negative values, the output BP signal is compatible with observations made on real data, e.g., the RC decay time constant is less than 10 s). This prior information is sufficient for correct inference in the simulation environment. However, it is possible that more complex priors will be needed when processing patient data.

Validation of this method is preliminary and limited for now to the simulation framework. The method cannot yet be applied to our patient data, as information on *d*, the arterial collapse value at brachial site (Equation (3)), is not available due to uncertainties regarding arm tissue compression and cuff compliance. This is currently being investigated as part of work conducted in parallel [15]. Nevertheless, the simulation framework does demonstrate that ample information regarding the hemodynamic status of a patient is contained within the distal BP response to cuff inflation and that opportunities for cuff-based measurement strategies should be explored further.

## 3. Results

### 3.1. Measurements

**Figure 4:**
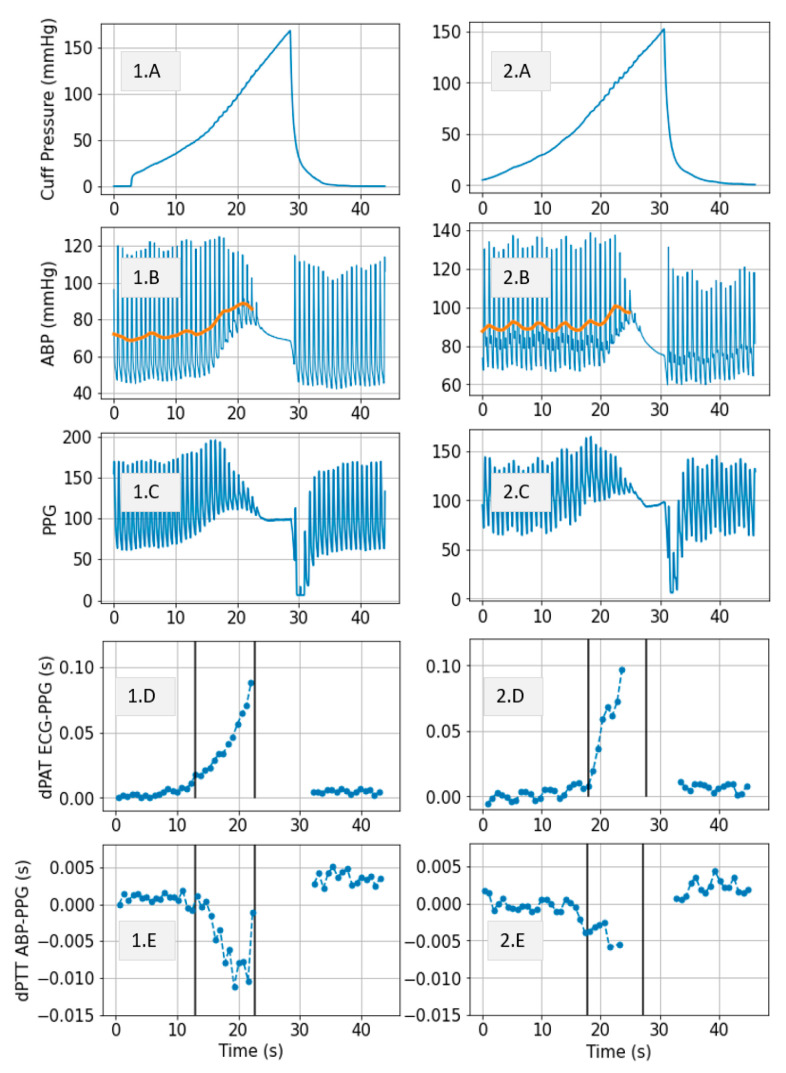
Example signals $Patient_{1}$ (**left**) and $Patient_{2}$ (**right**). Highly dynamic and patient specific effects are occurring in the distal arm as result of the cuff inflation. The plots show: (**A**) Cuff pressure; (**B**) Blue: Arterial pressure (ABP) measured invasively downstream form the cuff at radial site, Orange: MAP; (**C**) PPG signal measured at finger site downstream from the cuff; (**D**) $\Delta PAT_{ECG-PPG}\left(CuffP\right)$; (**E**) $\Delta PTT_{ABP-PPG}\left(CuffP\right)$.

To give a visual impression of the cuff inflation effects on distal arm arterial pressure, Figure 4(1A,2A) show a segment of the cuff pressure signal and Figure 4(1B,2B) show the simultaneously recorded ABP signal. Effects of cuff inflation on the distal limb BP have previously been assumed as irrelevant for characterization of pulse propagation along the arm [2]. However, our data shows that large BP changes occur due to cuff inflation.

Simultaneously recorded ECG and PPG are used to compute changes in PTT due to cuff inflation over two vascular segments: Heart to finger site $\Delta PAT_{ECG-PPG}\left(CuffP\right)$ (Figure 4(1D,2D) and radial to finger site $\Delta PTT_{ABP-PPG}\left(CuffP\right)$ (Figure 4(1E,2E)).

Our previous model [1,2] assumed that no significant changes in PTT occur in the distal arm during cuff inflation. Our current data however gives insights on the validity of this assumption. Figure 4(1E,2E) show a decrease in $\Delta PTT_{ABP-PPG}$ over the radial site to finger length of about 10 ms and 6 ms for for *Patient*_1_ and *Patient*_2_, respectively; therefore, the drop in ΔPTT over the entire length of the distal arm (defined as $\Delta PTT_{distal}$) is expected to be approximately ~20 ms and ~12 ms, respectively. 

**Figure 5:**
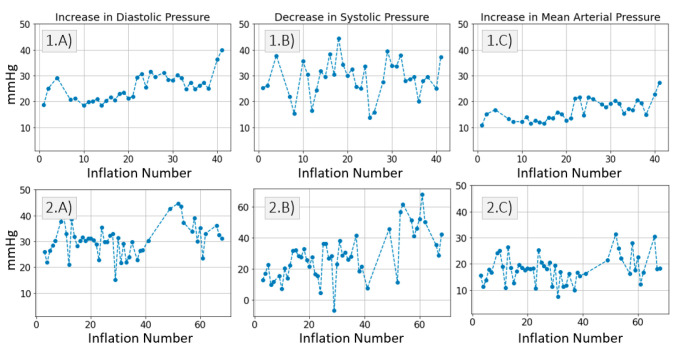
(**A**) Maximum increase in distal diastolic pressure per inflation. (**B**) Maximum decrease in distal systolic pressure per inflation. (**C**) Maximum increase in distal mean arterial pressure per inflation. Results are displayed for *Patient*_1_ data and *Patient*_2_ data respectively.

**Figure 6:**
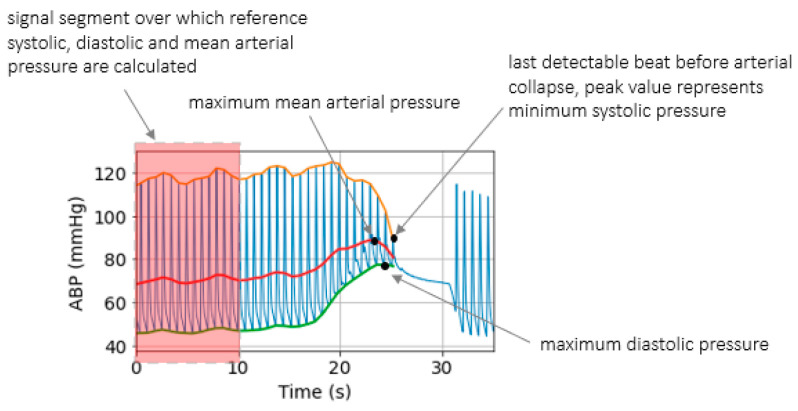
Illustration showing reference *P_sys_*, *P_dia_*, MAP.

Figure 5 summarizes the changes in distal BP across all inflations performed on *Patient*_1_ and *Patient*_2_, respectively. For each inflation, the maximum increase in diastolic pressure, the maximum decrease in systolic pressure and the maximum increase in mean pressure are calculated. Figure 6 illustrates how the reference BP values, maximum MAP, maximum *P_dia_* and minimum *P_sys_* are defined.

On average across all inflations performed on *Patient*_1_, the diastolic pressure increases by 25 mmHg, the systolic pressure decreases by 29 mmHg and the mean arterial pressure increases by 16 mmHg. On average across all inflations performed on *Patient*_2_, the diastolic pressure increases by 30 mmHg, the systolic pressure decreases 28 mmHg and the mean arterial pressure increases by 18 mmHg.

**Figure 7:**
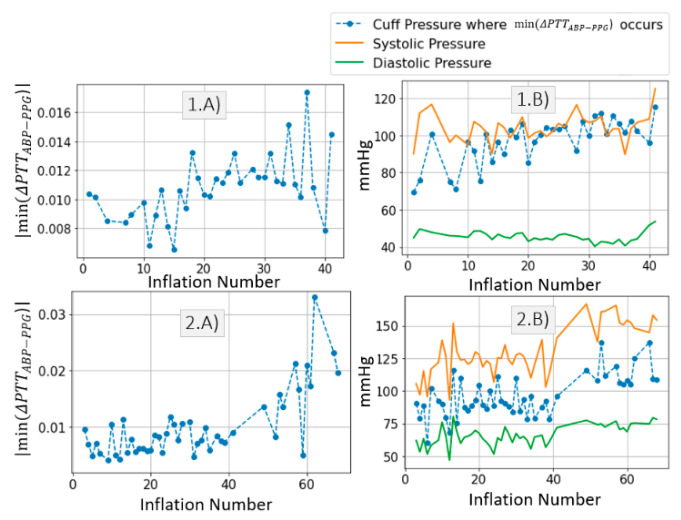
(**A**) Maximum drop in $\Delta PTT_{ABP-PPG}$ per inflation. (**B**) Cuff pressure at which the maximum drop in $\Delta PTT_{ABP-PPG}$ occurs, systolic and diastolic systemic values per inflation. Results are displayed for *Patient*_1_ data and *Patient*_2_ data respectively. Expected $\Delta PTT_{distal}$ is twice the value of $\Delta PTT_{ABP-PPG}$.

We quantify the decrease in $\Delta PTT_{ABP-PPG}$ (Figure 7(1A,2A) for all cuff inflations performed on *Patient*_1_ and *Patient*_2_, respectively. On average $\Delta PTT_{ABP-PPG}$ decreases by 10 ms for both patients. Standard deviation of $\Delta PTT_{ABP-PPG}$ values is 2.5 larger in *Patient*_2_ than in *Patient*_1_.

Figure 7(1B,2B) show the cuff pressure at which the maximum drop in $\Delta PTT_{ABP-PPG}$ occurs. It is found that the maximum drop in $\Delta PTT_{ABP-PPG}$ occurs when the cuff pressure increases beyond diastolic value. From this it can be deduced that the cuff inflation (and resulting distal BP increase) systematically lead to a drop in distal PTT. Therefore, the effect cannot be attributed to other factors.

### 3.2. Model Simulations and Inference Results

#### 3.2.1. Brachial Artery Compliance Inference

##### Simulation Framework 

**Figure 8:**
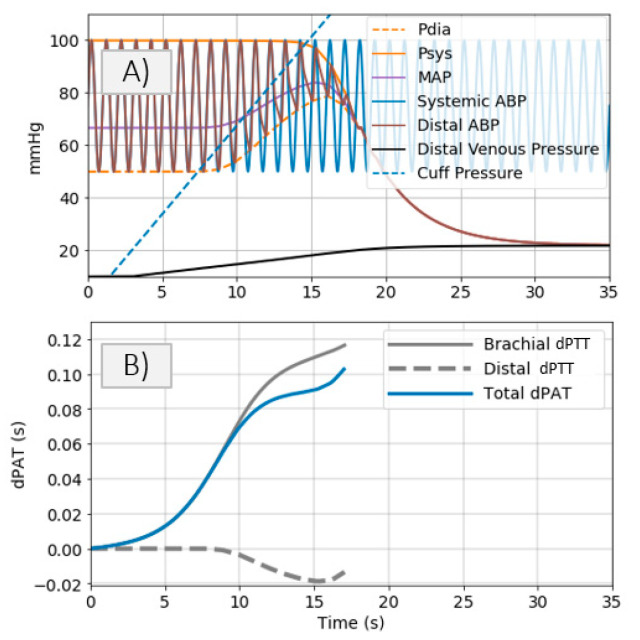
(**A**) Simulated systemic arterial pressure, cuff pressure and resulting distal arterial and venous pressure. (**B**) Simulated total Δ*PAT*, $\Delta PTT_{brachial}$, and $\Delta PTT_{distal}$.

The model shown in Figure 2 is used to simulate distal BP change with respect to cuff inflation. Figure 8A shows systemic arterial pressure, cuff pressure and resulting distal arterial and venous pressure. The simulated MAP increases by almost 20 mmHg during cuff inflation. This is similar to a typical change in MAP are observed in the patient data (Figure 4). The distal pulse transit effect is simulated (Figure 8B) by fitting a linear PWV-MAP relationship to obtain a $\Delta PTT_{distal}$ response to MAP increase similar to the observations on patient data (see Figure 4). Figure 8B also includes $\Delta PTT_{brachial}$ simulated with parameters *a* = 0.03 and *c* = 0.1 (as shown in [2]), and total Δ*PAT* (the summation of $\Delta PTT_{distal}$ and $\Delta PTT_{brachial}$).

**Figure 9:**
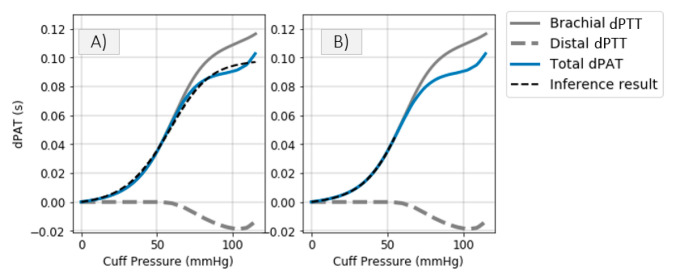
Inference of parameters *a* and *c* by (**A**) taking into account the entire dPAT recording, (**B**) discarding dPAT samples corresponding to cuff pressures greater than *P_dia_*.

To assess the impact of distal artery effects on the correctness of the inference process, the total dPAT is fitted as described in [2], under the assumption that $\Delta PTT_{distal}$ is not influenced by the cuff inflation. Figure 9A shows the fit result with a dotted black line. The inferred *a* value is 0.012 and the inferred *c* value is 0.08, corresponding to an error in *a* inference and *c* inference of over 50% and almost 20%, respectively. Therefore, changes in distal arm circulation are not negligible as assumed until now.

**Figure 10:**
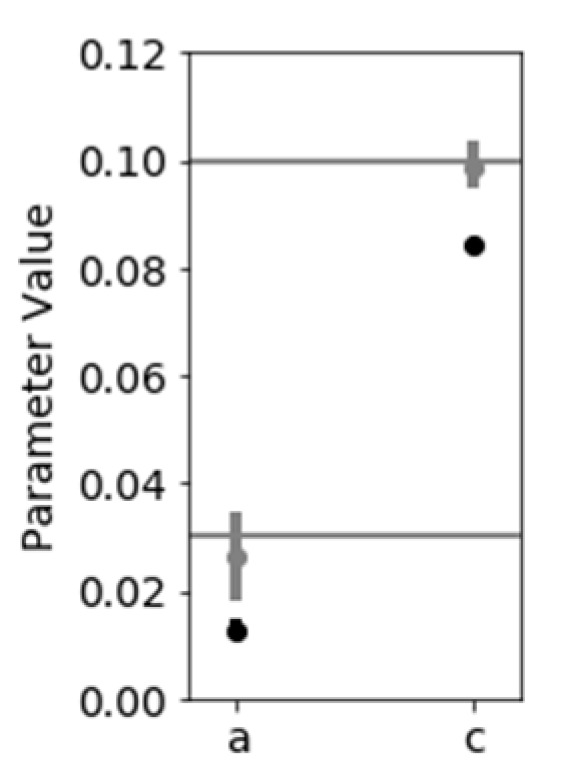
The parameter estimate are shown: the vertical bars indicate the 95% HDI. Black bars represent the HDI corresponding to the fit result shown in Figure 9A and gray bars represent the HDI corresponding to the fit result shown in Figure 9B.

A simple mitigation involves disregarding Δ*PAT* values recorded at cuff pressures greater than diastolic pressure. Simulations show that, in principle, it is possible to infer correct *a* and *c* values by taking into account the first portion of a Δ*PAT* recording only. Figure 9B shows the fit result with a dotted black line; the inferred *a* value is 0.027 and the inferred *c* value is 0.099, which are close to the true parameter values of 0.03 and 0.1, respectively. Figure 10 gives more information on the inference result by showing the 95% HDI, which contains the 95% most probable parameter values (more details in [2]). A larger uncertainty is present when disregarding Δ*PAT* data recorded at high cuff pressures.

Regarding our patient data, such small Δ*PAT* changes which occur in the first part of the inflation would ideally be recorded over much slower inflation speeds than we show in Figure 4. Alternatively, if an invasive line is available as in Figure 1, then the distal $\Delta PTT_{distal}$ can be accounted for.

##### Inference of Brachial Artery Parameters from Patient Data

**Figure 11:**
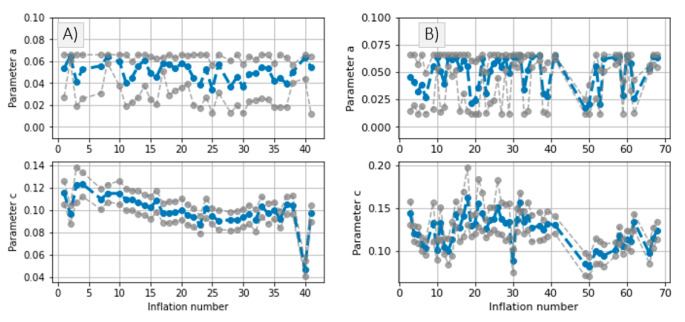
Inference results of the parameters *a* and *c* per inflation; (**A**) *Patient*_1_ data and (**B**) *Patient*_2_ data. Blue represents the central tendency of the posterior distribution, gray represents the 95% HDI.

The inference method is applied to our patient data as described in Section 2.3.1 The results are shown in Figure 11.

**Figure 12:**
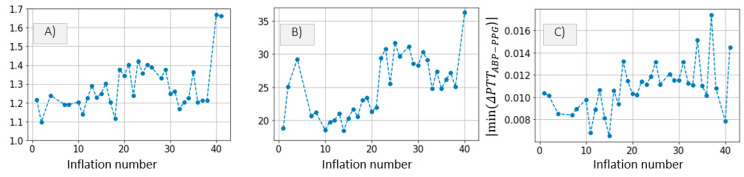
*Patient*_1_ data; (**A**) HR, (**B**) increase in *P_dia_* and (**C**) maximum drop in $\Delta PTT_{ABP-PPG}$ are plotted for qualitative comparison.

A slight correlation between parameter *c* and the parameters shown in Figure 12 (HR, maximum *P_dia_* increase and maximum distal ${\Delta \mathrm{PTT}}_{\mathrm{ABP}-\mathrm{PPG}}$ decrease) can be noticed. In *Patient*_1_, the *c* value decreases gradually up to inflation number ~5, after which a plateau is reached, followed by a slight increase. An opposite trend is observed in plots showing heart rate (HR), maximum *P_dia_* increase and maximum drop in $\Delta PTT_{ABP-PPG}$ (Figure 12).

**Figure 13:**
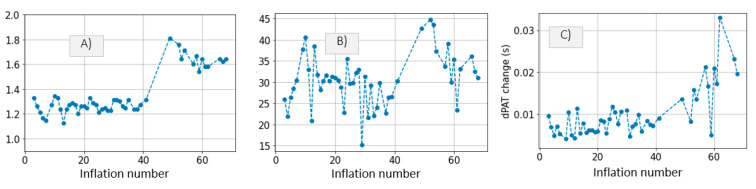
*Patient*_2_ data. (**A**) HR, (**B**) increase in *P_dia_* and (**C**) maximum drop in $\Delta PTT_{ABP-PPG}$ are plotted for qualitative comparison.

In *Patient*_2_, *a* drop in the *c* value is noticed at inflation 50. Again, the opposite trend is observed in HR, maximum distal *P_dia_* increase and maximum drop in $\Delta PTT_{ABP-PPG}$ (Figure 13). Such qualitative assessment suggests that the c parameter is to some extent linked to systemic changes in hemodynamic status.

#### 3.2.2. Estimation of PAT-BP Calibration

The inferred *a* and *c* parameter values (Figure 11) are used to obtain the BP-PAT calibration. By the method described in Section 2.3.2, the ECG and PPG signals are processed over a number of time segments to estimate beat-to-beat *P_sys_*. One time segment is defined as the duration between two consecutive cuff inflations.

**Figure 14:**
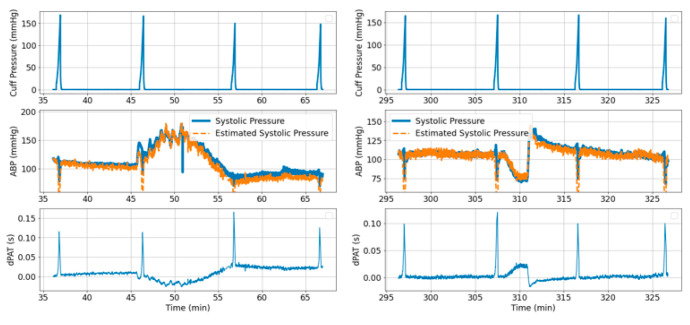
Example of PAT-based BP estimation in *Patient*_1_.

**Figure 15:**
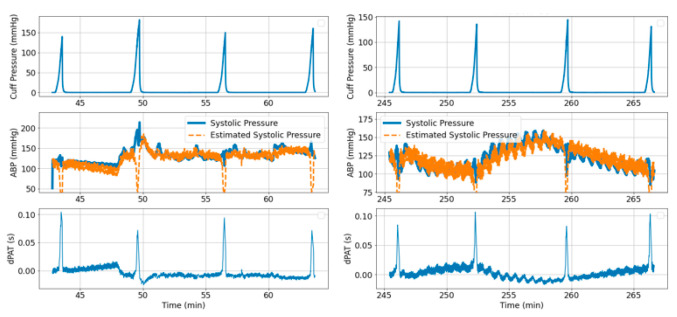
Example of PAT-based BP estimation in *Patient*_2_.

To illustrate the algorithm performance, Figure 14 and Figure 15 shows examples of BP prediction over a total of six time segments obtained from *Patient*_1_ and *Patient*_2_ data, respectively.

**Figure 16:**
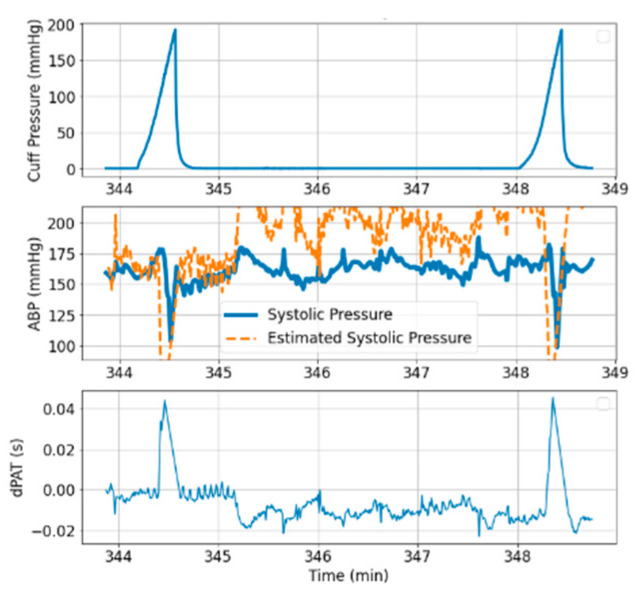
Example of time segment not included in the analysis.

Over the entire recording, we find the *P_sys_* estimation to be generally accurate. To define a performance criterium, *P_sys_* estimation is considered inaccurate when an error larger than 10 mmHg occurs over a duration longer than one minute. For *Patient*_1_, the algorithm estimates *P_sys_* inaccurately over two out of thirty-six time segments, and for *Patient*_2_, the algorithm estimates *P_sys_* inaccurately over five out of thirty-six time segments. Note that the PAT-based estimation of BP can only work when the pulse transit is undisturbed by motion. Figure 16 shows an example of a time segment where significant motion occurs due to the invasive nature of the procedure. For this reason, we did not account for 11 time segments from which breathing rate could not be distinguished (assuming that motion artifacts are minimal if breathing is expressed in the signal).

#### 3.2.3. Inference of Distal Arm Circulation Parameters

##### Time Constant and Mean Systemic Filling Pressure

**Figure 17:**
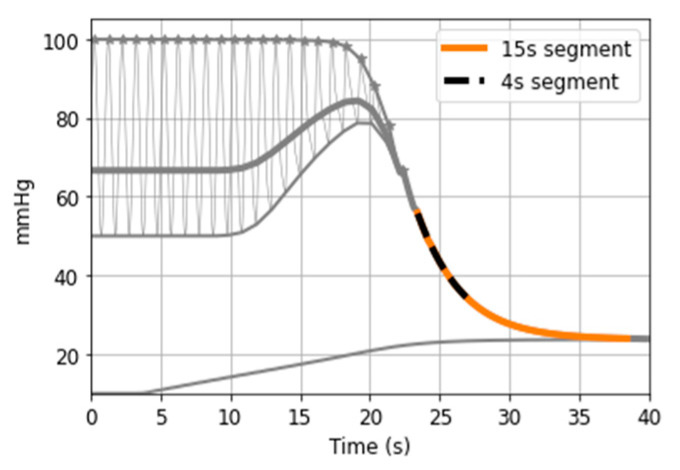
Feasibility of simulation-based time constant and $P_{Equilibrium}$ measurement.

A distal BP signal is generated with the same parameter values as in Section 3.2.1. Figure 17 illustrates the 15 s segment of the BP signal where the artery is fully collapsed. Analytically (according to the series combination of arterial compliance, systemic resistance and venous compliance), the time constant of the exponential decline is equal to 3.08 s and the $P_{Equilibrium}$ is equal to 23 mmHg (note that in this case $P_{Equilibrium}$ does not equal *P*_msf,_ because artery and vein do not collapse simultaneously). An exponential function is fitted to the selected segment and inferred values are τ = 3.18 s and $P_{Equilibrium}$ = 23 mmHg. This suggests that the inference method is feasible. However, our data contains fast inflations of about 30 s in total, with exponential decline segments of ~4 to 5 s long (Figure 4). Therefore, it is important to check if τ and $P_{Equilibrium}$ inference is possible when short recordings are available. Figure 17 highlights the first 4 s of the exponential decline segment. Fitting an exponential decline function to this short segment results in τ = 3.4 s and $P_{Equilibrium}$ = 22 mmHg.

**Figure 18:**
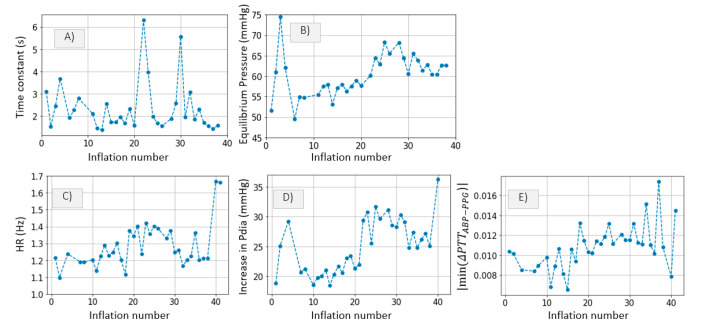
(**A**) Time constant τ and (**B**) $P_{Equilibrium}$ from *Patient*_1_ data. (**C**) HR, (**D**) increase in *P_dia_* and (**E**) maximum drop in $\Delta PTT_{ABP-PPG}$ are plotted for qualitative comparison.

Ideally, a longer cuff inflation is necessary to infer the time constant parameter accurately. Nevertheless, the algorithm is applied to our patient data in order to obtain an approximation of the parameter values. Figure 18A,B show the τ and $P_{Equilibrium}$ inference results for each of the 35 cuff inflations performed on *Patient*_1_. One clear trend is observed in the $P_{Equilibrium}$ inference results. The $P_{Equilibrium}$ value increases gradually up to inflation number ~25, after which a plateau is reached, followed by a slight decline. The same trend is observed in plots illustrating HR, maximum *P_dia_* increase and maximum distal $\Delta PTT_{ABP-PPG}$ decrease with respect to inflation number (Figure 16), suggesting that systemic changes in the hemodynamic status are occurring. A plausible explanation to this correlation is that HR determines the amount of blood which is pumped by the heart into the arm throughout the 30 s inflation. Therefore, when HR increases, blood fills up the arm vasculature at a faster rate, leading to larger changes in distal diastolic pressure prior to arterial collapse, and also leading to an increase in $\Delta PTT_{distal}$ drop and τ.

**Figure 19:**
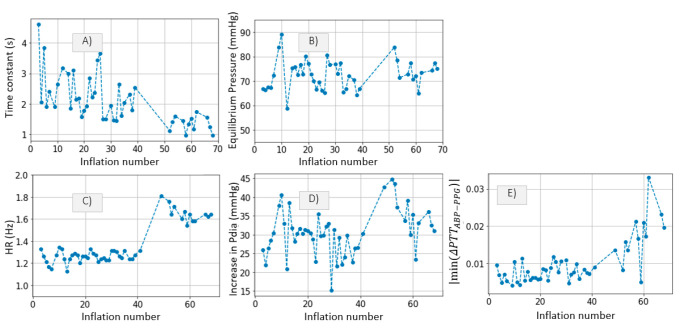
(**A**) Time constant τ and (**B**) $P_{Equilibrium}$ from *Patient*_2_ data. (**C**) HR, (**D**) increase in *P_dia_* and (**E**) maximum drop in $\Delta PTT_{ABP-PPG}$ are plotted for qualitative comparison.

Figure 19A,B show the τ and $P_{Equilibrium}$ inference results for *Patient*_2_. A decrease in τ is observed at inflation fifty. Significant changes in HR, maximum *P_dia_* increase and maximum drop in $\Delta PTT_{ABP-PPG}$ are also observed around inflation fifty. It is clear that at the time of inflation fifty, the hemodynamic status of *Patient*_2_ is altered. However, the correlation between hemodynamic variables is slightly different from *Patient*_1_. The change in the hemodynamic status is not clearly visible when analyzing the $P_{Equilibrium}$ values. A distinguishable change in τ, however, is observed at inflation fifty.

Such correlations are interesting to observe, and they indicate that τ and $P_{Equilibrium}$ are to some extent affected by systemic alterations in the circulatory system.

##### Systemic Resistance, Distal Vascular Compliance

The distal BP signal simulation (generated with the parameter values described in Section 3.2.1) is processed to infer distal arterial compliance, systemic resistance and venous compliance via the MCMC fitting method described in Section 2.3.3. The fit result, the HDI and central tendency of the resulting posterior distribution are shown in Figure 10.

The correct estimation of the model parameter values from the simulated BP signal demonstrates the feasibility of the inference method and that the BP response to cuff inflation can be processed to obtain information on systemic resistances, arterial and venous compliances.

## 4. Discussion

Interesting dynamic effects occur in the vasculature as response to occlusion-based perturbations. Our goal is to understand these effects in depth. The insights are applied towards developing cuff-based measurement strategies for acquiring information on a number of hemodynamic parameters of potential interest to critical care.

We previously studied the response of the vasculature to occlusion perturbations via non-invasive modalities. In this study, we advance our understanding by invasively measuring BP downstream from the cuff. We observe highly dynamic effects occurring in the distal arm as result of the cuff inflation. Figure 4 shows the extent to which distal BP is altered due to cuff inflation: $P_{dia}$ increases by 30 mmHg, MAP increases by 20 mmHg. This effect is also evidenced by the 20-ms drop in $\Delta PTT_{distal}$. The cuff-induced effects on distal vasculature are systematic and are seen in both investigated patients across all cuff inflations (Figure 6 and Figure 7).

A model is developed to obtain an understanding of the factors influencing the observed behaviors (Figure 2). Simulations conducted via the model with parameters within ranges close to values reported in the literature mimic the effects observed in the patient data. Five cuff inflation stages which are identified based on the patient data (Figure 4) are also present in the model output (Figure 2 upper right plot):

1. Cuff pressure value is below systemic venous pressure: arm circulation remains unaltered.
2. Cuff pressure increases beyond systemic venous pressure: vein collapses, flow out of the limb is stopped and buildup of blood begins to occur in the limb via the artery; arterial pressure is not visibly altered at this stage.
3. Cuff pressure increases beyond systemic diastolic pressure leading to increase in distal diastolic pressure.
4. Cuff pressure approaches systemic systolic pressure: minimal amount of blood flows into the limb at each heart-beat, a decrease in the distal systolic pressure is observed.
5. Eventually, blood flow is stopped—arterial and venous pressures tend towards an equilibrium value.

The model closely mimics all 5 stages of the cuff inflation process, meaning that the model can be used to represent changes in distal arterial pressure during cuff inflation and that physiological meaning can be attributed to the model parameters. With the help of the proposed model, we explore several measurement strategies.

### 4.1. Inference of Brachial Artery Compliance

Via a simulation framework we show that the inference of brachial parameters *a* and *c* is inaccurate when distal PTT effects are not accounted for (Figure 9 and Figure 10). Two mitigations are possible:

- Disregarding dPAT values recorded at cuff pressures greater than *P_dia_*. This leads to accurate inference of the parameters *a* and *c*. One downside is in that a relatively high degree of uncertainty is present (Figure 10). This method is not suited to our data, which includes fast inflation speeds and few PAT points recorded at cuff pressures below *P_dia_*.
- Alternatively, if an invasive line is available as in Figure 1, then the distal PTT can be accounted for. We apply this method to our data (Figure 11).

We observe some correlation between the parameter *c* and other hemodynamic parameters: HR, maximum distal diastolic increase and maximum distal dPTT decrease. Such qualitative assessment gives indication that the *c* parameter is to some extent linked to systemic changes in the hemodynamic status. The relatively large HDI of the parameter *a* inference result and the outlier parameter *c* value occurring at inflation 40 of *Patient*_1_ suggest that the measurement procedure needs to be further optimized, possibly via slower cuff inflation speeds, which can allow for the collection of more dPTT datapoints. Improved measurement devices with higher sampling rate, better PPG contact pressure will also likely enable better inference of parameters.

### 4.2. Estimation of PAT-BP Calibration

The inferred *a* and *c* parameter values (Figure 11) were used to estimate beat-to-beat *P_sys_* over time segments following each processed inflation (Figure 14 and Figure 15). We find the *P_sys_* estimation to be generally accurate over the majority of time segments. This gives indication that the inferred *a* and *c* parameter values are in the correct range. In 7 of the 72 time segments, the estimation is inaccurate. It is not yet clear which effects cause this temporary drop in performance. It is likely that the model (Figure 2) is not yet complete and that other dynamic mechanisms are taking place. The model also assumes a homogeneous artery segment, which might contribute to inference innacurracy. In addition, Equation (3) [6] which characterizes arterial collapse has been developed based on insights acquired via ex-vivo methods of research. High resolution imaging and specialised setups are needed to further validate the arterial collapse mechanisms in-vivo [7]. Parallel work is also tackling uncertainties regarding cuff, arm tissue, artery interaction [15,21].

Also, the presented PAT-based BP estimation is intended for patients that are not monitored invasively. The method might perform better during less invasive OR procedures, or during ICU monitoring, where hemodynamic alterations are expected to be less frequent and less abrupt, allowing for more accurate measurement of cuff-induced effects on the vasculature. Also, the absence of motion artifacts caused by invasive interventions would increase the reliability of the PAT-based BP estimation.

### 4.3. Inference of Distal Arm Circulation Parameters

#### 4.3.1. Time Constant and Mean Systemic Filling Pressure

We first investigate the RC decay BP segment following arterial collapse. This portion of the signal is influenced by the least amount of physiological effects, e.g., brachial artery collapse mechanics, and heart and lung activity does not affect the vasculature behavior at this stage. Via a simulation framework, we demonstrate the measurement of the time constant τ and $P_{Equilibrium}$ to be feasible. If the inflation speed is increased such that arterial and venous collapse occur simultaneously, then $P_{Equilibrium}$ would be identical to the mean systolic filling pressure *P*_msf_. Previously investigated cuff-based *P*_msf_ measurement modalities involve complete vascular occlusion of about ~30 s [19]. Our simulation framework shows preliminary results that this occlusion duration can be reduced to ~5 s if RC decay principles are taken into account.

The proposed algorithm is applied to the patient data and a qualitative assessment is performed. In *Patient*_1_, we observe a correlation between $P_{Equilibrium}$ and a number of other hemodynamic parameters: HR, distal *P_dia_* increase, maximum drop in $\Delta PTT_{ABP-PPG}$ and parameter *c* (Figure 18). However, in *Patient*_2_, we observe a correlation between τ and the rest of the hemodynamic parameters (Figure 19). This qualitative assessment of the correlations between the inferred parameters and the hemodynamic measurements gives some indication of the link between distal vasculature parameters and systemic changes in hemodynamic status. Further investigation is possible with dedicated clinical studies designed to assess possible links between changes in distal vasculature parameters and hemodynamic instability events.

#### 4.3.2. Systemic Resistance, Distal Arterial and Venous Compliance

A method accounting for all portions of the BP response to cuff inflation was developed in order to obtain information on arm vasculature resistance *R_s_*, distal arterial *C_a_* and venous compliance *C_v_*. It is evident that the physiological meaning is approximate—for example, it is possible that *R_s_* adapts to some extent throughout the inflation process. Therefore, $R_{s}$ can be perceived as an indicator of resistance, rather than as an exact measurement (this uncertainty applies also to the other parameters $C_{a}$, $C_{v}$, τ, $P_{Equilibrium}$, *a* and *c*, which might be affected by the cuff inflation itself). Further clinical evidence involving multiple patient groups can indicate if changes in arm vasculature resistance are linked to changes in systemic resistance and if there is a correlation between relative changes in parameters such as $C_{a}$, $C_{v}$ and subsequent changes in blood pressure, patient outcome etc. It is likely that factors such as type of procedure, level of sedation, or conditions such as arteriosclerosis, endothelial dysfunction impact vasculature response to cuff inflation.

**Figure 20:**
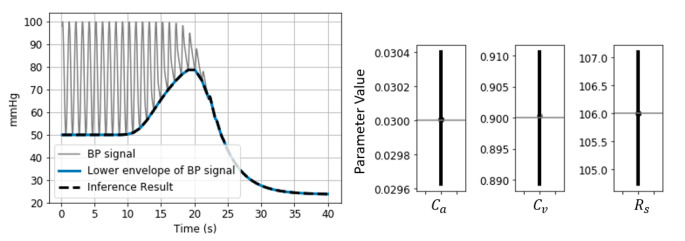
Simulated BP signal and inference result. The signal is simulated with control parameter values $C_{a}$ = 0.03 mL/mmHg, $C_{v}$ = 0.9 mL/mmHg, $R_{s}$ = 106 mmHg·s/mL. The inferred parameter values (determined as the central tendency of corresponding posterior distributions) are equal to the control parameter values.

In our study, the validation of the method is preliminary and is limited to the simulation framework (Figure 20). The method cannot yet be applied to our patient data, as information on the arterial collapse value at brachial site (Equation (3)), *d*, is not available due to uncertainties regarding arm tissue compression and cuff compliance. This is currently being investigated as part of work conducted in parallel [15]. Further development might be needed when applying the method to patient data. For example, a more informative prior might be necessary, due to the complex interference between multiple physiological effects influencing the signal.

Nevertheless, the proposed simulation framework does demonstrate that extensive information regarding the hemodynamic status of a patient is contained within the distal BP response to cuff inflation and that related opportunities for cuff-based measurement strategies should be explored further.

### 4.4. Outlook

The presented research method is intended to serve as a basis for further studies aimed at characterizing vasculature response to occlusion-based perturbations. Several strategies aimed at modulating blood flow and pulse propagation need to be further investigated: stepwise/continuous inflation/deflation, venous vs. arterial occlusion, varying duration of occlusion, inflation speed, frequency of occlusions, length over which occlusion is applied, site at which occlusion is applied.

In addition to this, studies which address occlusion-related effects via non-standard measurement setups might contribute to the development of improved models. For example, interesting pathophysiological mechanisms are being observed via NIRS-based measurement of tissue oxygenation response to vascular occlusion tests [22] and cuff devices are being used for evaluation of endothelial function and nitric oxide regulation [23].

Many other emerging techniques which measure interactions between hemodynamic variables in response to defined perturbations will also facilitate further exploration of cuff-based measurement setups [24].

## 5. Conclusions

This study improves our understanding of vasculature response to occlusion-based perturbations. Initial characterizations [1,2] neglected the changes occurring in the distal limb. However, our new experimental evidence shows that highly dynamic processes occur in the distal vasculature during cuff inflation. A distal arm model and a simulation framework were developed based on experimental evidence in order to explore cuff-based modulation of blood flow and pulse propagation along the artery.

We demonstrate new possibilities to interpret the cuff-induced changes and obtain information of potential value to critical care: PAT-BP calibration, brachial arterial compliance, distal vascular compliance, peripheral resistance, mean systemic filling pressure, artery-vein interaction. Feasibility of the measurements was mainly demonstrated via computer simulations and via qualitative assessment of limited patient data; this work is meant as basis for further clinical studies.

## Data Availability

Restrictions apply to the availability of the data due privacy regulations.
